# Temperature Effects on Gametophyte Life-History Traits and Geographic Distribution of Two Cryptic Kelp Species

**DOI:** 10.1371/journal.pone.0039289

**Published:** 2012-06-18

**Authors:** L. Valeria Oppliger, Juan A. Correa, Aschwin H. Engelen, Florence Tellier, Vasco Vieira, Sylvain Faugeron, Myriam Valero, Gonzalo Gomez, Christophe Destombe

**Affiliations:** 1 Center for Advanced Studies in Ecology and Biodiversity, Facultad de Ciencias Biologicas, Pontificia Universidad Católica de Chile, Santiago, Chile; 2 UPMC Station Biologique de Roscoff, Equipe Biologie Evolutive et Diversité Marine, “BEDIM”, UMR 7144, BP 74, Roscoff, France; 3 CNRS Station Biologique de Roscoff, Equipe Biologie Evolutive et Diversité Marine, “BEDIM”, UMR 7144, BP 74, Roscoff, France; 4 Centre of Marine Sciences (CCMAR), CIMAR – Laboratório Associado, Universidade do Algarve, Campus de Gambelas, Faro, Portugal; 5 Departamento de Ecología, Facultad de Ciencias, Universidad Católica de la Santísima Concepción, Concepción, Chile; Rutgers University, United States of America

## Abstract

A major determinant of the geographic distribution of a species is expected to be its physiological response to changing abiotic variables over its range. The range of a species often corresponds to the geographic extent of temperature regimes the organism can physiologically tolerate. Many species have very distinct life history stages that may exhibit different responses to environmental factors. In this study we emphasized the critical role of the haploid microscopic stage (gametophyte) of the life cycle to explain the difference of edge distribution of two related kelp species. *Lessonia nigrescens* was recently identified as two cryptic species occurring in parapatry along the Chilean coast: one located north and the other south of a biogeographic boundary at latitude 29–30°S. Six life history traits from microscopic stages were identified and estimated under five treatments of temperature in eight locations distributed along the Chilean coast in order to (1) estimate the role of temperature in the present distribution of the two cryptic *L. nigrescens* species, (2) compare marginal populations to central populations of the two cryptic species. In addition, we created a periodic matrix model to estimate the population growth rate (λ) at the five temperature treatments. Differential tolerance to temperature was demonstrated between the two species, with the gametophytes of the Northern species being more tolerant to higher temperatures than gametophytes from the south. Second, the two species exhibited different life history strategies with a shorter haploid phase in the Northern species contrasted with considerable vegetative growth in the Southern species haploid stage. These results provide strong ecological evidence for the differentiation process of the two cryptic species and show local adaptation of the life cycle at the range limits of the distribution. Ecological and evolutionary implications of these findings are discussed.

## Introduction

Understanding the mechanisms that limit geographical distributions of species has long been a key question in both ecology and evolutionary biology [1], [2] and it is generally accepted that multiple causes (both biotic and abiotic) can be interacting. Variations of geographic species range resulting from current climatic change have been widely demonstrated, including, for example, poleward movements of species’ range boundaries in fish [3], mammals [4], birds [5],butterflies [6], and seaweeds [7]. Predictions of range shift are generally based on statistical relationships between the current species distribution and selected environmental variables. However, physiological limitations can constrain the distribution ranges and abundance of organisms [8]. There is a clear need for improved understanding of how the variation of environmental factors in space and time affect critical fitness components such as survival and reproduction [2], [9].

To study the mechanisms that constrain a species’ distribution, a highly informative zone is the edge of the range itself [10], [11], particularly to study evolutionary processes. First, because marginal populations tend to occur in patches, genetic drift is expected to be stronger than in central populations where distribution is continuous [12]. As a consequence, marginal populations are expected to be genetically deprived, leading to a higher sensitivity to environmental changes in comparison with central populations. Furthermore, when dispersal is too low, even small abiotic variations in space and/or in time may have a large impact on the persistence of these local populations [12]. However, marginal populations may also be a place where local adaptation occurs, due to the particular environmental conditions, although this process depends on the relative genetic isolation of the marginal populations and on the species’ dispersal capacity.

Temperature is considered as the most important factor determining the geographic distribution of numerous species, as it affects survival, reproduction and/or growth [13]. This is particularly true for benthic marine macroalgae [14]. Kelps (Laminariales, Phaeophyceae) are mainly cold temperate species occurring from polar to inter-tropical zones. They play a major ecological role by structuring the ecosystem and are commercially exploited for alginate extraction [15]. At low latitudes, their range edge is generally determined by warm temperatures and nutrient limitation (see for review Steneck et al. 2002). In the tropics, kelps are restricted to deep-water cool habitats [16], [17] confirming the effect of temperature on kelp distribution.

A quantitative estimate of multiple fitness components across the life cycle is necessary to understand the mechanisms that define the range limit [18], [19]. Because of their complex life cycle, kelps are an especially interesting model. These species display a heteromorphic life history with an alternation of microscopic haploid gametophytes and diploid sporophytes [20] so that their range limit may be determined by the cryptic gametophytic stage. This microscopic stage is quite impossible to observe *in situ* but easy to cultivate in the laboratory from spores compared to the macroscopic sporophytic stage issued from wild individuals. Thermal responses of macroscopic sporophytes and microscopic stages are generally consistent with the geographical distribution of species and strongly depend on the species studied (macroscopic stage: [14], [21]; microscopic stages: [22]–[26]). Tom Dieck [27] hypothesized that the distribution of the five different *Laminaria* species in the Northern Atlantic is following a gradient from cold-temperate to warm-temperate microscopic stage adaptation. In addition, Matson and Edwards [28] suggested that difference in temperature response of the microscopic stages could explain the difference in the location of the southern limit of range distribution between *Pterygophora californica* (less tolerant to warm temperature) and *Eisenia arborea* along the North Eastern Pacific coast. Similarly, the range distribution of two Ecklonia species across the cold-to- warm transition of the southern tip of South Africa is positively associated with optimal temperature for growth and fertility of the gametophyte stage [29]. However, very little is known about the variation of response of the microscopic stage to temperature among kelp populations across their entire geographic range.

The South Eastern Pacific temperate coast is particularly interesting to study the effects of temperature fluctuation, because Sea Surface Temperature (SST) shows a complex pattern of both spatial and temporal variability. While a general trend of increasing temperatures with decreasing latitude is described in the Humboldt Current System [30], a patchy structure of thermal conditions along the coast is created by upwelling centers where cold, nutrient-rich subsurface waters are upwelled by equator-ward winds [30]. In addition, temporal fluctuations of SST are occurring: (i) at inter-annual scales due predominantly to El Niño Southern Oscillation (ENSO) events, (ii) at the seasonal scale, and (iii) at the synoptic scale (several days), associated to the alternation between high and low atmospheric pressure and rain, mostly in temperate areas, and (iv) at the daily scale for intertidal species, associated to the tidal regimes [31], [32]. In comparison to southern regions, the northern part of the Humboldt Current System is dominated by permanent anticyclonic conditions but occasionally strongly affected by ENSO events [30]. At a smaller scale, the marine biogeographic transition zone described around 30°S of latitude in the Chilean coast seems to have unpredictable but high temperature fluctuations at inter-annual scales at the north of 30°S, in contrast to predictable and limited temperature fluctuations at intra-annual scales south of 30°S [31]. Consequently, fluctuating temperatures and the duration of exposure to stress must be considered along with mean temperatures present in the coast.

**Figure 1:**
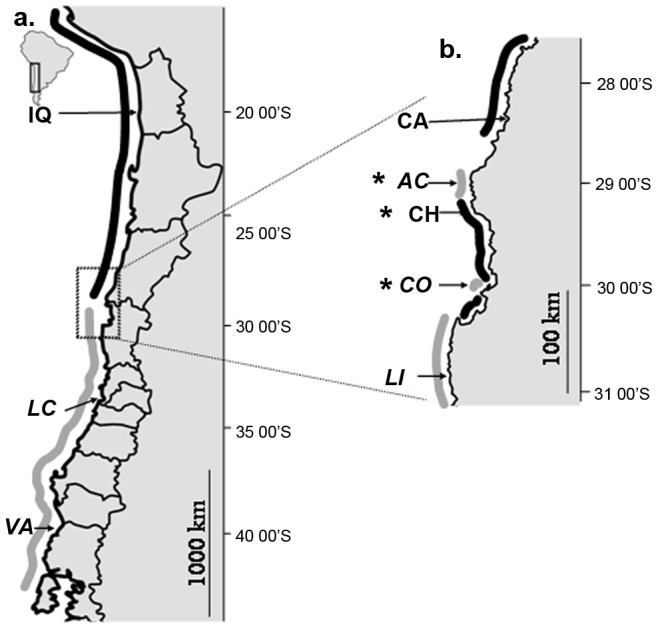
Distribution of Northern and Southern cryptic species of *Lessonia nigrescens*: (a) along the Chilean coasts, (b) detail of the transition zone (28–31°S). The range distribution of the Southern species is represented in grey (names in italics) and of the Northern species in black. Marginal populations are indicated by an asterisk. See Table 1 for details.

We chose to study the case of the intertidal kelp *Lessonia nigrescens* for which two cryptic species have been recently identified along the South-eastern Pacific coast [33]. These two kelp species have contrasting geographic ranges: the ‘Northern species’ occurs between 16°S and 30°S and the ‘Southern species’ stretches between 29°S and 41°S (Fig. 1). Further studies have shown that these two species are reproductively isolated [34] and were never found co-existing in the same location, even within the transition zone between the two range distributions (from 29°S to 30°14′S), where a mosaic of pure populations either of the Northern or Southern species was observed whatever the scales [33], [35]. Because of the contrasting distribution ranges, the species experience different environmental conditions such as water temperature with the Northern species occurring in warmer waters than the Southern species. They are also differentially exposed to environmental disturbances. For example, during the El Niño event of 1982/83, a massive mortality affected individuals from the northernmost populations of the Northern species, nevertheless in the affected region, some populations survived in certain localities, such as Iquique (20°S, [36]). It has been hypothesized that this survival could have been the result of local adaptation to high temperatures [36], [37].

Using these two cryptic species of the *Lessonia*, we aimed to test the following hypotheses regarding the tolerance to temperature stress of microscopic stages: (i) the Northern species is expected to be more tolerant to high temperature than the Southern species, and (ii) local adaptation within each species is expected as a differential thermal tolerance among populations across geographic range. Particularly, we expected that marginal populations, located in the transition zone, would present singular responses to temperature.

**Table 1:** Names and positions of the sampling locations along the Chilean coast, ordered from north to south.

Location	Abbreviation	Species	Type	Latitude	Longitude	Sampling date
Iquique	IQ	Northern	Central	20°25'62”S	70°12'48”W	05-05-2008
Carrizal Bajo	CA	Northern	Central	28°04'27”S	71°08'36”W	05-05-2008
Chañaral de Aceituno	AC	Southern	Marginal	29°04'03”S	71°29'26”W	03-06-2008
Choros Ventana	CH	Northern	Marginal	29°12'57”S	71°28'23”W	03-06-2008
Coquimbo-Cruz	CO	Southern	Marginal	29°57'15”S	71°21'44”W	15-06-2008
Río Limarí	LI	Southern	Central	30°44'10”S	71°42'05”W	06-05-2008
Las Cruces	LC	Southern	Central	33°30'09”S	71°38'01”W	06-05-2008
Valdivia	VA	Southern	Central	39°46'75”S	73°23'49”W	04-06-2008

## Methods

### Site Characterization and Sampling

Fertile sporophytes were sampled in eight intertidal locations along the Chilean coast from 20°25′S (Iquique) to 39°46′S (Valdivia, Fig. 1, Table 1). Sampling effort was especially focused on the overlapping transition zone of the two cryptic species, corresponding to the geographical range margin of each species. The other locations of both the Northern and the Southern species were considered thereafter as central populations. The two species are morphologically similar and thus were distinguished based on length polymorphism of the atp8/trnS mitochondrial marker as described in Tellier et al. [33], [34]. Satellite records of Sea Surface Temperatures (SST) were used as a proxy of water temperature for all the study near shore sites. SST were estimated from long time survey data (25 years, 1982–2007) of the Advanced Very-High Resolution Radiometer (AVHRR) satellite [38]. SST ranged from 11.0°C (monthly mean minima) to 15.2°C (monthly mean maxima) in Valdivia and respectively from 16.2°C to 23.0°C in Iquique. Temperature in the transition zone ranged from 13.2°C to 17.9°C. We considered SST differences between sites to be robustly estimated since comparison between *in situ* loggers (located between 28° and 36°S) and AVHRR estimations of SST showed a significant correlation (0.83<r^2^<0.96) according to locations with a slope-value close to 1, (Tapia, com. pers.) evidencing a slight but constant overestimation of the satellite estimations, independently of the temperature.

### Experimental Design, Spore Release and Culture Conditions

15 fertile fragments of sporophytic individuals from each location were grouped in three sub-sets of five individuals each. For each subset and each treatment condition, we induced spore release and initiated cultures in two replicate 50 mL Falcon tubes (BD Biosciences, San Jose, CA, USA) following the methods of Oppliger et al. [35]. Five temperature treatments were chosen to include the temperature range experienced by the species in natural conditions (see *Site characterization*), with three fixed temperature conditions: 10°C, 15°C and 20°C. In addition, to consider the temporal variation of the environmental conditions, we included two variable temperature regimes: 10–15°C and 15–20°C, where the culture temperature was changed every three days (initiating the culture at the lowest temperature, i.e. 10°C and 15°C respectively). This protocol corresponded to a total of 240 culture tubes: 5 temperature treatments applied to 8 locations, each composed of 3 individual subsets, with two replicates tubes.

### Characteristics of the Life Cycle and Criteria for Life History Stages Determination

The genus *Lessonia* exhibits a heteromorphic haploid-diploid life cycle with an alternation of microscopic haploid dioecious gametophytes and macroscopic diploid sporophytes (several meters long) that produce haploid spores by meiosis. Male and female gametophytes show clear sexual dimorphism. Female gametophytes produce eggs that, after fertilization by sperm, produce new diploid sporophytes. Previous studies suggested that the dispersal capacity of both cryptic species is limited [33], [34].

**Figure 2:**
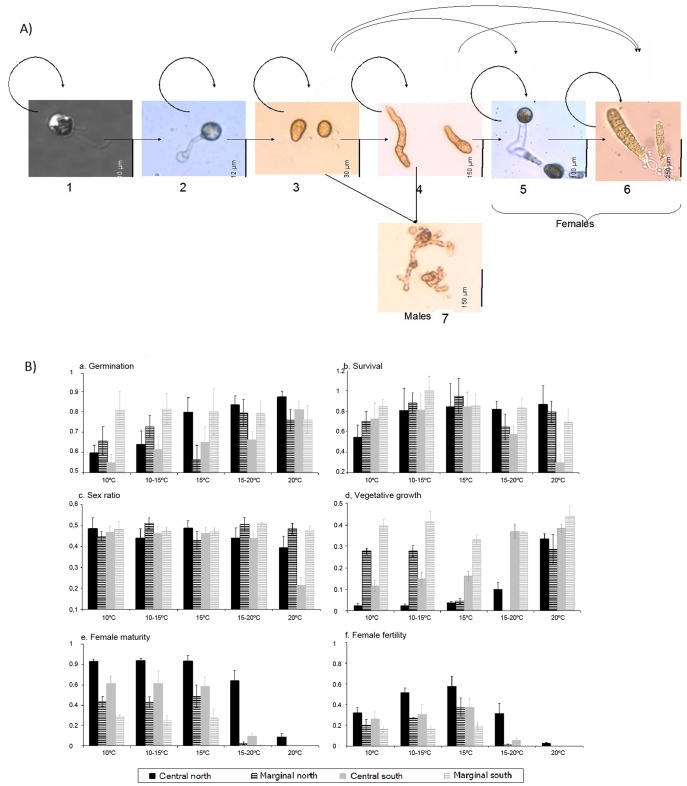
Microscopic stages and population growth results for the Northern and Southern cryptic species of the *Lessonia nigrescens*. (A) The gametophyte life history diagram considered in this study. Arrows show transitions within and between the distinguished stages: (1) spores, (2) germling spores, (3) gametophyte of 1–2 cells, (4) gametophyte of >2 cells, (5) mature female, i.e. with oogonia, (6) fertilized females, bearing a young sporophyte, and (7) male gametophyte. (B) Mean vital rates (± SE) for each of the four defined groups of populations (central populations of the Northern and Southern species, and marginal populations of the Northern and Southern species) of *Lessonia nigrescens* cryptic species, at each of the five temperature treatments. a. Germination rate (at day 2 of culture), b. Gametophyte survival (day 25), c. Sex ratio of gametophytes, i.e. the frequency of male gametophytes (day 15), d. Frequency of female gametophytes showing vegetative development, i.e. having more than 2 cells (day 24), e. Female maturity rate, i.e. frequency of females with oogonia (day 25), f. Female fertilization rate, i.e. frequency of females bearing a sporophyte (day 25).

Seven microscopic life-history stages were defined (Fig. 2A), using a combination of developmental characteristics and reproductive structures of the gametophytes [20]: 1) settled meiospores, 2) germinated spores, identified by the formation of a protuberance that becomes a germination tube, 3) gametophytes of 1–2 cells, 4) gametophytes of >2 cells, 5) reproductive female gametophytes, i.e. bearing oogonia (female fecundity), 6) fertilised female gametophytes, i.e. bearing microscopic sporophyte (female fertility), 7) male gametophytes. Since formation of oogonia has been observed in one-celled, two-celled and multicellular gametophytes of *Lessonia* [39], we distinguished two types of female gametophytes: 1–2 cells and >2 cells, to estimate the effect of culture conditions on the frequency of vegetative growth before reaching maturity. Male and female gametophytes were unambiguously identified because of their important sexual dimorphism: male gametophytes are narrower than female gametophytes and display highly branched filaments formed by small cells (Fig. 2A).

### Life History Traits Estimations and Data Analyses

Observations were made on days 2, 5, 13, 15, 17, 24, 25 of culture. All life history stages (as previously defined, see also Fig. 2A) were counted on three marked visual fields per slide using a Nikon Eclipse TE300 inverted microscope (Nikon Corp., Tokyo, Japan) using 10X objective, except for the germination estimate (20X objective). For each observation date, survivorship was estimated by counting the number of well-colored gametophytes over the initial count (day 2 of culture) on the same area. The germination rate was estimated at day 2 and 5 of culture by counting the number of germinated spores over the total number of spores that were alive. At days 13, 17 and 24 of culture, the frequencies of female gametophytes of 1–2 cells and of female gametophytes of >2 cells were determined to characterize vegetative development. Sex ratio was estimated after 15 days in culture and was expressed as the frequency of males, i.e. males/(males+females). Finally, reproduction was estimated at days 13, 17 and 24 of culture: 1) the frequency of mature females was estimated over 30 females in each visual field, and 2) female fecundity was estimated indirectly using the frequency of females bearing juvenile sporophytes in each visual field.

Hypothesis 1 (comparison between Northern and Southern species) was tested through comparison of central populations of both species, i.e. the populations located within the continuous range of each species (2 locations for Northern species: Iquique and Carrizal Bajo and 3 locations for Southern species: Río Limarí, Las Cruces and Valdivia). The fixed model used was: species, treatment and treatment*species, with the factors species (2 levels) and temperature treatment (5 levels). Hypothesis 2 (comparison between marginal and central populations) was tested only for the Southern species (2 marginal locations: Chañaral de Aceituno and Coquimbo; 3 central locations: Río Limarí, Las Cruces and Valdivia) using a 2-way ANOVA with marginality (fixed, 2 levels: central and marginal) and temperature treatment (fixed, 5 levels) as factors. Alternatively we performed a two-way ANOVA with the factors location (fixed, 3 levels) and temperature treatment (fixed, 5 levels). Differences among locations were considered in relation to marginality differences in the Southern species. The response variables were the life history estimates: rates of germination, survival, vegetative development, sex ratio, female maturity and female fecundity. Data was transformed (rank or square root (arc sin X) transformation, as recommended for proportion data [40], and examined for normality and homogeneity of variances. Type III sum of squares were used for tests of significance. All statistical analyses were done with MINITAB v. 13.2 (State College, PA, USA), by performing general linear models and Tukey’s tests for *a posteriori* multiple comparisons (*α* = 0.05).

### Periodic Matrix Model

The matrix model was structured into six stage classes as previously defined, but excluding the male gametophytes for stages 5 and 6 because of difficulties in recognizing male reproductive structures (Fig. 2A). Transition probabilities among stage classes were estimated by calculating the proportion of individuals of each stage that transited to another stage for selected observation intervals, resulting in periodic matrices for each census intervals (days 0–2, 2–5, 5–13, 13–17 and 17–24). As the purpose of the experiment was to elucidate temperature dependent effects on the development of microscopic stages, the entire sporophytic diploid part of the life cycle was reduced to a fecundity vector describing the spore production of each microscopic stage over its entire life: [0 1 10 100 1,000 10,000]. As for a matrix approach the life cycle of the organism needs to be closed (cyclic), it was necessary to assign a fecundity vector to all microstages as in some combinations of locations x temperatures gametophytes did not (all) reach the microscopic sporophyte phase by the end of the experiment. This vector was used for all treatment combinations equally and means that spores (stage 1) produce no spores, that germinated spores (stage 2) produce 1 spore and that the final stage 6 produces 10,000 spores.

For each location and temperature treatment, the population dynamics was described by a non-linear projection matrix A, where the element aij represents the probability of transition from stage j to stage i over one census period. The growth of each population was projected by multiplying the transition matrix with a column vector *n_t_*, which includes the number of individuals in each stage class at time t:
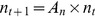
(1)


The dynamics of each population over the cycle was described by the periodic matrix produced by multiplying all matrices (*B*) of a location x temperature combination, sequentially [41], [42]:
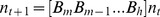
(2)

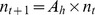
where the periodic cycle starts at census period *h* and ends at period *m*. In this way, for each combination of location and temperature, a model was created which consisted of a data set of five matrices (one for each observation interval) containing transitions. The population growth rate (fitness equivalent) was calculated as the dominant eigen value (λ) of the product of each set of matrices. We estimated the population growth rate for each population * treatment condition. Uncertainties in the population growth rate were estimated from bootstrap confidence intervals (95%) by using the percentiles of the distribution of 1,000 bootstrap estimates. No bias adjustment or bias estimation was implemented, because these only reduce certain kinds of bias while greatly reducing the precision of the resulting estimates [43].

Hypothesis 1 (comparison between Northern and Southern species) and 2 (comparison between marginal and central populations within each species) were tested for the integrative variable of growth rate. These tests followed the same model than for the vital rates described previously. The response variables were the growth rate estimates using the mean value of the 1,000 bootstraps estimates. Data was transformed (fourth root (X) transformation) to fit the assumptions for normality and homogeneity of variances.

Elasticities identify the most vulnerable or important transitions of a life history, that is the transitions which have a greater effect on the population fitness (or growth rate, [44], [45]). The elasticity values of each observation interval were estimated by calculating 5 periodic matrices, for the entire cycle beginning in each observation interval [46]. Since elasticities sum to one, each elasticity value may also be interpreted as the relative contribution of each matrix element to the population fitness [41], [44]. Thus, elasticities may be summed across selected regions of a matrix, corresponding to different demographic stages or processes, in our case in order to compare the relative importance of each stage class.

## Results

The results of the six mean vital rates are given for marginal and central populations of each species at each of the five temperature treatments (mean vital rates Fig. 2B and statistical analysis Table 2). Germination rates of central locations were higher for the Northern species than for the Southern species, independently of temperature (Fig. 2B). In both species, germination rate increased with temperature (no significant temperature*species interaction). In the Southern species, the germination rate was higher in marginal than in central populations at all but the 20°C treatment. Within the Northern species no differences were detected among locations (Table 2, Tukey test 10°C = 10–15°C <15°C = 15–20°C = 20°C).

**Table 2:** Results of 2-way ANOVA (fixed factors) for each of the six considered vital rates**.** d.f.: degree of freedom.

Vital rate		Germination		Survival		Sex ratio			Vegetative growth		Female maturity		Female fertility	
Groups of populations compared and sources of variation	d.f.	F	p	F	p	F		p	F	p	F	p	F	p
***a. Comparison between Northern and Southern species (central locations only)***														
Temperature	4	13.68	**<0.001**	0.58	0.674	11.71	**<0.001**		9.86	<0.001	38.13	**<0.001**	24.06	**<0.001**
Species	1	11.89	**0.001**	0.06	0.807	4.94	**0.028**		13.41	0.001	37.76	**<0.001**	45.16	**<0.001**
Temperature * Species	4	1.53	0.196	4.57	**0.002**	3.29	**0.013**		1.18	0.329	2.60	**0.039**	3.73	**0.007**
***b. Southern species: comparison between marginal and central locations***														
Temperature	4	3.64	**0.008**	4.95	**0.001**	8.59		**<0.001**	2.08	0.093	20.65	**<0.001**	13.76	**<0.001**
Marginality	1	6.58	**0.011**	5.55	**0.020**	20.92		**<0.001**	10.94	**0.002**	28.60	**<0.001**	9.98	**0.002**
Temperature * Marginality	4	1.05	0.386	0.10	0.984	8.41		**<0.001**	1.36	0.256	3.57	**0.008**	1.15	0.334
***c. Northern species: comparison among locations***														
Location	2	2.90	0.061	1.19	0.311	1.31		0.276	4.17	**0.025**	61.98	**<0.001**	63.32	**<0.001**
Temperature	4	8.63	**<0.001**	3.49	**0.011**	0.55		0.696	11.75	**0.000**	22.89	**<0.001**	20.99	**<0.001**
Temperature * Location	8	1.85	0.080	3.78	**<0.001**	1.27		0.274	4.86	**<0.001**	5.73	**<0.001**	5.00	**<0.001**

Bold type indicates significant differences at *α* = 0.05.

The survival rate varied significantly between species only for the higher temperature tested. At 20°C the survival of the Southern species central (i.e. cold-waters) populations dropped to 0.29, whereas the survival of the Northern species remained constant under this condition (Fig. 2B). Interestingly, marginal populations of the Southern species showed higher survival at 20°C than central populations (Table 2).

Although sex ratio generally remained close to 0.5, the frequency of males of the Southern species central populations dropped to 0.22 at 20°C (Fig. 2B). This effect was not observed in the Northern species or in the Southern species marginal populations.

The proportion of gametophytes exhibiting vegetative growth differed greatly between central populations of each species and between central and marginal populations. Entry into vegetative growth increased with temperature in central populations of both the Northern and Southern species; however gametophytes of the Southern species consistently exhibited greater vegetative development across all temperatures tested (Fig. 2B). There was a significant effect of marginality on the entry of gametophytes into vegetative development in the Southern species (Table 2), where Southern species marginal populations showed high incidences of gametophyte vegetative growth at all temperatures tested (Table 2). The single Northern species marginal population tested showed a more complex pattern of gametophyte vegetative growth with temperature. However, the response to temperature was also significantly different from that of the Northern species central populations (Table 2), showing high incidence of vegetative growth at treatments <15°C (Fig. 2B).

The maturity and the fertility of female gametophytes were different between species and varied according to the temperature (Fig. 2B). Maturity and fertility were higher in Northern species than in Southern species (Fig. 2B). These vital rates diminished dramatically in all populations at the higher temperature treatments tested (15–20°C and 20°C) (Fig. 2B). Only the Northern species central populations showed the ability to reproduce at 20°C. The marginal Southern populations exhibited consistently lower frequencies of female maturity and female fertility than the central populations (Fig. 2B).

**Figure 3:**
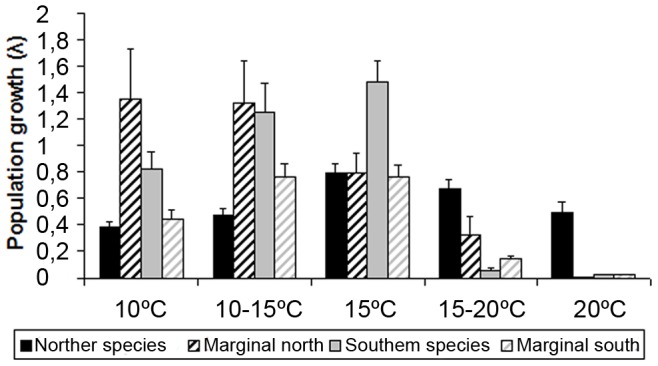
Mean population growth (± SE) for central and marginal populations from both species of *Lessonia nigrescens* under the five tested temperature treatments.

**Figure 4:**
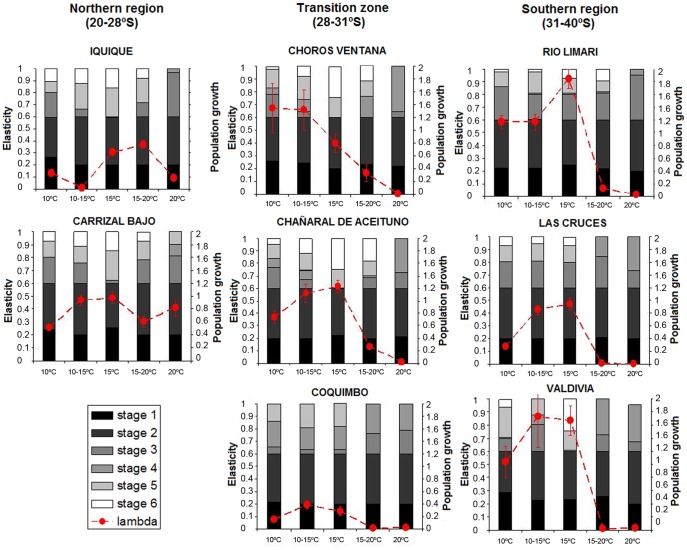
Mean population growths (± SE, lambda) and elasticities of the different transition elements of the matrices for each treatment for each studied population. Populations are ordered from left to right according to their geographic distribution: northern to 28°S, within the transition zone (i.e. marginal populations) and southern to 31°S).

The population growth rate calculated for central populations of both species showed differences face to treatments (Fig. 3; Table 3). Southern species central populations growth rates were consistently ca. 2-fold greater than those of the Northern species central populations for all but the highest temperature treatments tested, in which it dropped to nearly 0. In contrast, the population growth rate calculated for the Northern species central populations did not show as strong variation with temperature, remaining moderate at all temperature treatments and reaching a maximum of 0.79±0.07 at 15°C (interaction temperature * species; Table 3). Marginal Southern populations did not differ in population growth rate when compared to Southern species central populations (Table 3). Yet, populations within the Southern species showed differences between locations (data not shown; two-way ANOVA, F_4,16_ = 7.88, P = 0.001; Fig. 4). This is due to the location of Coquimbo that showed extremely low growth rates values for all temperature treatments (>0.2). No differences between Northern species populations were detected (Table 3).

Elasticity analyses revealed that stages 1 and 2 (spore and germination) contributed the most to the fitness of all populations (Fig. 4). In more advanced stages (stage 3 to stage 6) it was possible to observe differences between species (Fig. 4). The Northern species displayed an almost non-existent stage 4 (vegetative growth) and considerable elasticities for stages 5 and 6 (female fecundity and female fertility) for all tested treatments of temperature (except for 20°C), whereas the Southern species displayed important elasticities for stages 3 and 4 (vegetative growth), and less for 5 and 6 (female fecundity and female fertility). The marginal Northern and Southern populations exhibited considerable contribution of stage 4 compared to their respective central populations (Fig. 4).

**Table 3:** Results of 2-way ANOVA (fixed factors) for the population growth rates (lambda) variable.

Growth rate	lambda (λ)		
*Groups of populations compared*	d.f.	*F*	*p*
***a. Comparison between Northern and Southern species (central locations only)***			
Temperature	4	4.89	**0.010**
Species	1	0.30	0.591
Temperature * Species	4	6.19	**0.004**
***b. Southern species: comparison between marginal and central locations***			
Temperature	4	17.40	**<0.001**
Marginality	1	1.89	0.190
Temperature * Marginality	4	0.70	0.601
***c. Northern species: comparison among locations***			
Location	2	1.43	0.294
Temperature	4	0.56	0.698

Significant *p*-values are indicated in bold.

## Discussion

This study demonstrates that the microscopic stages of the two cryptic species of *Lessonia* show consistent differences in thermal responses and in life history strategies. In contrast to the Northern species, the Southern cold-waters species is not able to survive or to reproduce at high temperatures. These results suggest that, as expected, the Northern species is tolerant to higher temperatures than the Southern one, and that the geographic distribution of these two species seems to be related to the environmental conditions. Relationships between thermal responses and the location of distribution range boundaries have been shown previously in different macroalgal species [14], [47], [48]. A recent distributional shift of southern cold water species northwards was documented in the Northeastern Atlantic by Lima et al. [49]. Van den Hoek [50] demonstrated a strong association between temperature and seaweed range distribution and suggested that the southern and the northern boundaries of each species were dependent on three main effects: the temperature effect on survival (corresponding to the ‘lethal boundary’), on growth (‘growth boundary’), and on reproduction (‘reproductive boundary’). Van den Hoek [50] explained the difference of distribution between the two kelps *Laminaria digitata* and *Saccharina latissima* (as *Laminaria saccharina*) by the fact that *L. digitata* could be limited to the south by the ‘southern reproductive boundary’ of the gametophyte (corresponding to the 10°C February isotherm), while *S. latissima* could be limited by the ‘southern lethal boundary’ of the sporophyte (corresponding to the 19°C August isotherm). Recently, Viejo et al. [51] and Araújo et al. [52] demonstrated that marginal populations of the brown alga *Fucus serratus* showed a dramatic reduction of reproductive capacity when compared to central locations. Our study suggests a clear ‘reproductive northern boundary’ for the gametophytes of the Southern species that was unable to produce gametes at 20°C, even if the gametophytes were exposed only intermittently to 20°C (treatment 15–20°C). This phenomenon could explain the absence of the Southern species in the Northern region. Temperature could also explain the absence of the Northern species in the Southern area, because the Northern species showed the ability to grow and reproduce at low and high temperature treatments, but at treatments of 10°C, 10–15°C and 15°C the Southern species displayed higher populations growth rate that the Northern species, and this latter would be outcompeted by the Southern species in the south. These characteristics support the suggestion based on phylogeny that the Northern species originates from the Southern region [33]. Following the hypothesis developed by Breeman [14] to explain latitudinal range expansions in macroalgae, the Northern species might have conserved its ancestral capacity to grow and reproduce under low temperatures, and the colonization of the northern region might have been associated with an adaptation to warmer conditions [33]. This study demonstrates that the clear cut distribution of these two species observed along the Chilean coast can be explained by the differential response of the gametophytes to temperature.

The Northern and Southern species showed different life history traits. Gametophytes of the Northern species were generally small and usually consisted of one cell that immediately formed oogonia whereas gametophytes of the Southern species exhibited a higher vegetative development (of multiple cells) before forming oogonia. Consequently, female gametophytes from the Northern species develop rapidly but give very few oogonia per gametophyte, whereas female gametophytes from the Southern species delay their maturation, grow vegetatively and then produce numerous oogonia per individual. In a previous study done within the Southern species of *L. nigrescens* [53], patterns of development were observed to vary with temperature. Intraspecific plasticity was also observed in our study, yet the differences between Northern and Southern species were much stronger and consistently observed across the temperature ranges tested.

The influence of disturbance and climatic risks on the evolution of life span have been previously described in angiosperms [54]–[57], in agreement with theoretical predictions on the evolution of life history traits [58], [59]. For example, Hautekèete et al. [60] suggested that life span of the sea beet (*Beta vulgaris* ssp. *maritima*) may be explained by climatic factors and by habitat stability. Indeed, in this species, life history traits varied (life span and age at first reproduction) between populations. Populations with the longest-lived individuals occurred in the most stable habitats (Northern Europe) whereas populations with the shortest-lived individuals occured in the most disturbed locations (Southern Europe). In disturbed locations, a strong association between earlier reproduction and a short life span was demonstrated [53], [60]. Similarly, the higher reproductive output observed in marginal populations of *Ascophyllum nodosum* was assumed to be due to a life-history strategy that lead to population persistence in unfavourable environmental conditions [52]. In our study, we hypothesize that the life history of the Northern species might minimize exposure of the gametophyte stage to unpredictable environmental conditions (e.g. ENSO events) by earlier reproduction rapidly producing the larger sporophyte stage. In contrast, the Southern species is located in a more stable and colder environment, and delayed maturation might favour a long life span and allow completion of sexual reproduction when plants are more vigorous. Consequently, more time can then be spent on juvenile growth, which may be advantageous because larger gametophytes can produce more offspring [61]–[62].

Finally, for the estimated vital rates, marginal populations in this study showed a higher diversity of responses compared to core populations. Theoretically, marginal populations are expected to be more fragmented and more affected by genetic drift processes, leading to a higher differentiation between them than between central populations [2], [12]. Our results are therefore supporting the hypothesis that the differential response results from independent evolutionary processes.

While temperature tolerance has commonly been regarded as a conservative trait in many seaweeds [17], some studies have demonstrated ecotypic differentiation for this trait e.g. [14], [63], [64]. In our study, intraspecific differences of temperature tolerance were detected between marginal and central populations. For example, in both species, female maturity and fertility were consistently higher in central than in marginal populations. This is supported by differences in growth rates found between populations inside the Southern species. Yet, for the Northern species no differences were detected between populations. Interestingly, similar population growth rates were observed between geographically close populations across species (i.e. Chañaral de Aceituno and Choros Ventana), providing more evidence for the occurrence of local adaptation to temperature in populations at the range limits. Ecotypic variation in tolerance to high and/or low temperatures has been shown in kelps. For example, high-temperature tolerance observed in the sporophytes of *Saccharina latissima* (as *Laminaria saccharina*) in a southern marginal population in the North-East Atlantic was interpreted as genetic ecotypic differentiation in response to high-temperature [65]. In the same way, Martinez [37] pointed out the existence of different ecotypes in *L. nigrescens*, by studying thermal tolerance in the early sporophytic progeny from plants of different Chilean localities: now known to be occupied by Northern or Southern cryptic species. At the higher temperature tested, central and northern thermal ecotypes had higher survival and growth rates than the ecotypes from the south. At lower incubation temperatures the growth trend was opposite.

*Conclusions*. Our study of the microscopic stages of the two cryptic species of the *Lessonia* revealed different thermal tolerances between the two species, the Northern species being more tolerant to high temperatures than the Southern species. These results are congruent with the geographic distribution of the cryptic species, where higher temperature conditions exist in the northern region compared to southern area. In addition, the cryptic species showed different reproductive strategies that could be related to the stability of the environment. Furthermore, these results clearly highlight the importance of present day environmental discontinuity in shaping the distribution of species at this biogeographic boundary. Comparative studies of thermal tolerance are required to generalize our findings and further test the hypothesis that, even though the biogeographic transition seems to have an ancient origin [33], there are currently strong environmental determinants of discontinuity of many species distributions at 30°S.

Compared to animals, for which physiological parameters have been the main focus of attention to estimate ranges of tolerance to environmental heterogeneity and their effects on species distributions [8], we have shown here that modifications of the life cycle are important traits to analyze in algae. The shortening of the gametophytic phase for the Northern species opens new questions about the demographic characteristics of the alternation of generation and the role of life history trait evolution in the adaptation to different thermal conditions. In particular, it will be necessary to compare the life history traits of the sporophytic phase for the Northern and the Southern species to complement the present study.
