# Correction: Temperature Effects on Gametophyte Life-History Traits and Geographic Distribution of Two Cryptic Kelp Species

**DOI:** 10.1371/journal.pone.0110939

**Published:** 2014-10-13

**Authors:** The PLOS ONE Staff

In the Funding section, the grant number from the funder FONDECYT is listed incorrectly. The correct grant number is: FONDECYT 1090742.
